# Níveis de Interleucina-35 em Pacientes com Doença Arterial Coronariana Estável

**DOI:** 10.36660/abc.20200945

**Published:** 2021-12-08

**Authors:** Ersan Oflar, Mustafa Hakan Sahin, Bulent Demir, Abdulcelil Sait Ertugrul, Didem Melis Oztas, Metin Onur Beyaz, Murat Ugurlucan, Fatma Nihan Turhan Caglar

**Affiliations:** 1 Bakirkoy Dr Sadi Konuk Training and Research Hospital Departamento de Cardiologia Istambul Turquia Bakirkoy Dr Sadi Konuk Training and Research Hospital, Departamento de Cardiologia,Istambul - Turquia; 2 Akdeniz Saglik Yasam Vakfi Departamento de Cardiologia Antália Turquia Akdeniz Saglik Yasam Vakfi, Departamento de Cardiologia,Antália - Turquia; 3 Turkish Hospital Departamento de Cardiologia Doha Qatar Turkish Hospital, Departamento de Cardiologia,Doha - Qatar; 4 Bagcilar Training and Research Hospital Departamento de Cirurgia Cardiovascular Istambul Turquia Bagcilar Training and Research Hospital, Departamento de Cirurgia Cardiovascular,Istambul - Turquia; 5 Istanbul Medipol University Faculty of Medicine Departamento de Cirurgia Cardiovascular Istambul Turquia Istanbul Medipol University Faculty of Medicine, Departamento de Cirurgia Cardiovascular,Istambul - Turquia

**Keywords:** Aterosclerose, Doença da Artéria Coronariana, Interleucina-35, Pontuação de Propensão (Escore Gensini, Escore Syntax

## Abstract

**Fundamento:**

Foi demonstrado que as subunidades de interleucina-35 (IL-35) estão fortemente expressas nas placas ateroscleróticas em humanos. Assim, considera-se que elas têm um papel na aterosclerose.

**Objetivos:**

Neste estudo, os níveis de IL-35 foram comparados com o grupo controle em pacientes com doença arterial coronariana (DAC) estável, e a associação entre os níveis de IL-35 e o tipo, gravidade e extensão da lesão foram investigadas com o escore Gensini (GS) e o escore Syntax (SS) no grupo de pacientes

**Métodos:**

Sessenta pacientes (18 mulheres e 42 homens) com DAC, diagnosticados por meio da angiografia coronária, que apresentaram dor no peito típica e teste de esforço não invasivo positivo, e 46 pacientes (18 mulheres e 28 homens) com luminograma normal, foram incluídos no estudo. Tanto o GS quanto o SS foram calculados para o grupo de pacientes, e esses valores foram comparados com os níveis de IL-35. Variáveis com distribuição não normal foram avaliadas com o teste U de Mann-Whitney, enquanto os parâmetros com distribuição normal foram analisados com o teste t de Student. A diferença entre as variáveis categóricas foi avaliada pelo teste de qui-quadrado ou de Fisher. Os valores de p<0,05 foram considerados como estatisticamente sinificativos.

**Resultados:**

Não foram observadas diferenças significativas entre pacientes e o grupo controle em termos de características demográficas e achados laboratoriais. Em comparação ao grupo controle, os níveis de IL-35 no grupo com DAC foram consideravalmente menores (36,9±63,9 ng/ml vs. 33,2±13,2 ng/ml, p<0,008). Embora não tenha sido estatisticamente significativo, os níveis de IL-35 foram maiores em pacientes com SS mais baixo do que nos com SS mais alto (33,2±13,7 vs. 31,8±8,9, p=0,51). Os valores de IL-35 em pacientes com GS alto foram significativamente mais baixos do que em pacientes com GS baixo (35±17,4 vs. 30,7±8,6, p=0,043).

**Conclusão:**

Demonstrou-se que os níveis de IL-35 podem ser um novo biomarcador para a DAC estável, e que a IL-35 está associada à extensão da DAC.

## Introdução

A doença arterial coronariana (DAC) é uma das principais causas de morte pelo mundo, embora a prevalência de mortalidade por DAC tenha recentemente apresentado queda na Europa e nos Estados Unidos.^1,2^ A DAC é uma disfunção progressiva e sistêmica causada pela aterosclerose.^3^

Embora a inflamação tenha papel importante no desenvolvimento da aterosclerose, o mecanismo que a causa não está claro até o momento.^4^ As citocinas anti-inflamatórias, como a interleucina-10 (IL-10), e o fator de crescimento transformante beta tipo 1 (TGF-1) são amplamento avaliados nos ensaios sobre aterosclerose.^5-11^ Estudos publicados recentemente demonstraram que enquanto os níveis baixos de IL-10 e TGF-1 estão associados à progressão da aterosclerose e ao desenvolvimento da síndrome coronariana aguda, níveis altos de IL-10 e TGF-1 estão correlacionados ao bom prognóstico na DAC.^5-11^

A interleucina-35 (IL-35), uma citocina anfi-inflamatória recentemente definida, suprime a atividade das células CD4+T, induz a produção de células T regulatórias e reduz a progressão de doenças inflamatórias e autoimunes; por isso, tem um papel importante na aterosclerose.^12-15^

O objetivo deste estudo foi avaliar os níveis plasmáticos de IL-35 em pacientes com DAC estável e a associação entre IL-35 e a gravidade e extensão da DAC utilizando os escores Syntax (SS) e Gensini (GS).

## Métodos

Este é um estudo transversal e observacional realizado em um centro de referência terciário. Cento e seis pacientes consecutivos, que foram submetidos à angiografia coronária diagnóstica no Bakırköy Dr. Sadi Konuk Research and Training Hospital – Clínica de Cardiologia, entre julho de 2018 e maio de 2019, foram incluídos no estudo. O grupo de pacientes consistia de 60 pacientes (42 homens e 18 mulheres) com DAC estável que apresentaram estenose arterial coronária epicárdica de mais de 50% na angiografia coronária (AGC). O grupo controle consistia de 46 pacientes (28 homens e 18 mulheres) com dor no peito, mas AGC normal. Todos os pacientes tinham sido submetidos à avaliação objetiva da isquemia modificada, e todos tiveram resultados positivos em relação à isquemia. O número necessário de pacientes foi decidido com base em estudos prévios.^7^ Os pacientes adequados para o estudo de acordo com os critérios de inclusão foram incluídos até atingirmos o número necessário de indivíduos entre julho de 2018 e maio de 2019.

O diagnóstico prévio de diabetes melitus (DM), o uso de remédios para diabetes ou o nível de glicose em jejum de 126 mg/dl em dois momentos, em pacientes previamente não tratados, foram necessários para diagnosticar a DM. A hipertensão (HT) foi definida com base no uso prévio de medicações para hipertensão, pressão sistólica maior que 140 mmHg, ou pressão diastólica maior que 90 mmHg em pelo menos dois momentos separados. A taxa de filtração glomerular (TFG) foi estimada utilizando a equação da Modificação de Dieta em Doença Renal (MDRD) na internação. O Índice de Massa Corporal (IMC) foi calculado de acordo com os critérios da Organização Mundial da Saúde (OMS). A hiperlipidemia (HL) foi definida com base no uso prévio de antilipêmicos nos seis meses anteriores, ou altos níveis de lipídios séricos medidos após 8 horas de jejum [lipoproteína de baixa densidade (LDL) >160 mg/dl, colesterol total (CT) >240 mg/dl, ou triglicérides (TG) >160 mg/dl)].

Todos os pacientes assinaram um termo de consentimento, e este estudo está de acordo com a Declaração de Helsinki. Nossa pesquisa foi aprovada pelo Conselho de Ética Institucional.

Pacientes com DAC diagnosticada, TFG estimada (TFGe) <60ml/min, aqueles com doença valvar, indivíduos com pressão arterial sistólica maior que 140 mmHG e pressão arterial diastólica maior que 90 mmHg apesar do tratamento, insuficiência cardíaca, insuficiência renal, infecção aguda/crônica, febre, dores musculares, dores de cabeça, indivíduos em uso de antibióticos, indivíduos com doença imunoproliferativa, doença reumática, câncer, osteoporose e aqueles com mais de 75 anos foram excluídos.

### Avaliação objetiva da isquemia

Todos os pacientes tinham sido submetidos a um teste de esforço não-invasivo para a avaliação da isquemia. Na maior parte dos casos, o protocolo de Bruce modificado foi realizado. Uma depressão de segmento ST de pelo menos 1 mm, horizontal ou decrescente, em derivações ≥2 após 60*80 milissegundos desde o ponto J durante o exercício, foi considerada anormal. O escore de Duke foi usado para a estratificação do risco.^11^ Uma AGC foi realizada em pacientes que tinham escore de Duke médio ou alto. Uma cintilografia do miocárdio perfusão (CMP) foi realizada para avaliar a isquemia em pacientes internados com bloqueio atrioventricular de primeiro grau ou depressão de ST ≥1mm no eletrocardiograma de repouso (ECG), teste de esforço não diagnóstico, ou com pouca capacidade de esforço. A AGC foi realizada em pacientes que apresentavam nível de isquemia médio ou alto na CMP.

### Medidas de biomarcadores

Todos os dados laboratoriais dos pacientes, como troponina cardíaca T (TnT), creatinina, contagem de leucócitos, proteína C reativa de alta sensibilidade (PCR-as) etc., foram documentados.

Amostras sanguíneas para IL-35 foram coletadas no laboratório de cateterização antes da angiografia coronária em todos os participantes. As amostras foram obtidas por punção venosa com uso de EDTA (ácido etilenodiaminotetracético), em tubos sem aditivos, e imediatamente centrifugadas a 4.000 rpm por 10 minutos. As amostras foram coletadas após a centrifugação e armazenadas a -80ºC até a análise (não mais do que 6 meses).

As amostras foram descongeladas somente uma vez. A técnica do sanduíche, duplo anticorpo à base de biotina, utilizando o kit ELISA, foi usada para a análise de IL-35 (interleucina 35 humana: Yehua Biological Technology; Cat No:YHB1739Hu). A IL-35 foi adicionada a poços pré-revestidos com o anticorpo monoclonal IL-35, e, depois, incubada. Após a incubação, os anticorpos anti-IL-35 identificados com biotina foram adicionados à unidade com estreptavidina-HRP para formar o complexo imune. Após a lavagem, as enzimas não ligadas foram removidas e, depois, adicionadas aos substratos A e B. A solução tornou-se azul e transformou-se em amarelo com o efeito do ácido. Os tons da solução e a concentração de IL-35 estiveram positivamente correlacionados. Os resultados foram expressos em ng/ml. Os coeficientes de variação (CV) intra e entre ensaios da análise foram de <10% e <12%, respectivamente.

### Angiografia coronária

Todos os procedimentos de AGC foram realizados com o técnica de Judkins, por meio de um procedimento de cineangiografia (Axiom Artis, Siemens, Alemanha). Todas as angiografias foram registradas em *compact discs* (CDs), no formato DICOM, e visualmente examinadas por dois cardiologistas intervencionistas experientes e cegos para o estudo. A gravidade e extensão da DAC foram avaliadas de acordo com os SS e GS. O nível de estreitamento do lúmen, a concentricidade e a excentricidade foram avaliados. De acordo com o GS, 1 ponto é dado para 1-25% de estenose; 2 pontos, de 26-50%; 4 pontos, de 51-75%; 8 pontos, de 76-90%; 16 pontos, de 91-99%; e 32 pontos para 100%. Depois, o número de pontos para cada lesão é multiplicado pelo coeficiente considerado para cada segmento vascular principal, de acordo com a significância funcional do vaso (artéria coronária principal esquerda x 5; segmento proximal da artéria coronária descendente anterior esquerda x 2,5; segmento proximal da artéria circunflexa x 2,5; segmento médio da artéria coronária descendente anterior esquerda x 1,5; artéria coronária direita, segmento distal da artéria coronária descendente anterior esquerda, artéria posterolateral e artéria obtusa marginal x 1; e outros x 0,5), e a soma de todos os pontos constitui a pontuação total.^16^ O escore foi realizado com dois observadores e o valor médio. GS < 20 foi considerado como DAC leve (grupo 1), e GS ≥ 20 foi considerado como DAC grave (grupo 2). A SS correspondendo à complexidade da lesão foi medida pelas características da árvore coronária, assim como a localização da lesão e suas especificidades.^17^ O escore é medido por meio de uma calculadora virtual de acesso livre (www.syntaxscore.com). A pontuação foi realizada e a média foi calculada por dois observadores, cegos aos grupos do estudo.

### Análise estatística

A análise estatística foi realizada com o software SPSS, versão 16. A distribuição normal das variáveis contínuas foi analisada por métodos visuais (historama) e analíticos (Kolmogorov-Smirnov). As variáveis contínuas com distribuição normal foram demonstradas como média±desvio padrão (DP). Variáveis contínuas com distribuição não normal foram demonstradas como mediana e intervalo interquartil. As variáveis de distribuição não normal foram avaliadas com o teste U de Mann-Whitney, enquanto os parâmetros de distribuição normal foram analisados pelo test t de Student não pareado. As diferenças entre as variáveis categóricas foram avaliadas pelo teste de qui-quadrado ou de Fisher. A associação entre variáveis de distribuição não normal foi analisada pelo teste de Spearman; por outro lado, o teste de Pearson foi usado para a correlação entre as variáveis de distribuição normal. O teste de qui-quadrado foi usado para a sensibilidade, especificidade, valores preditivos negativos e positivos. A análise de regressão logística foi realizada para demonstrar a IL-35 como um fator de risco independente da DAC entre os fatores de risco tradicionais. A efetividade e a compatibilidade do modelo criado foram verificadas com o teste de Hosmer-Lemeshow. Valores de p <0,05 foram considerados como estatisticamente significativos.

## Resultados

**Figura 1:**
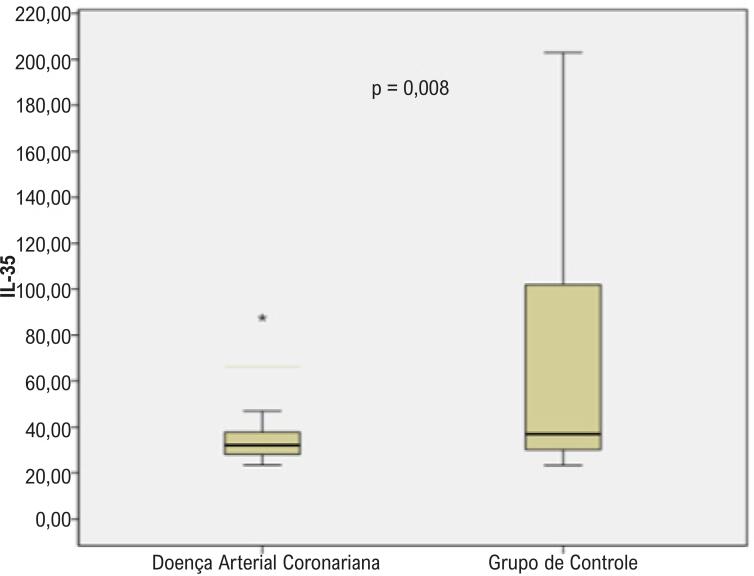
– Níveis de IL-35 nos grupos de pacientes e controles (Box-plot).

**Figura 2:**
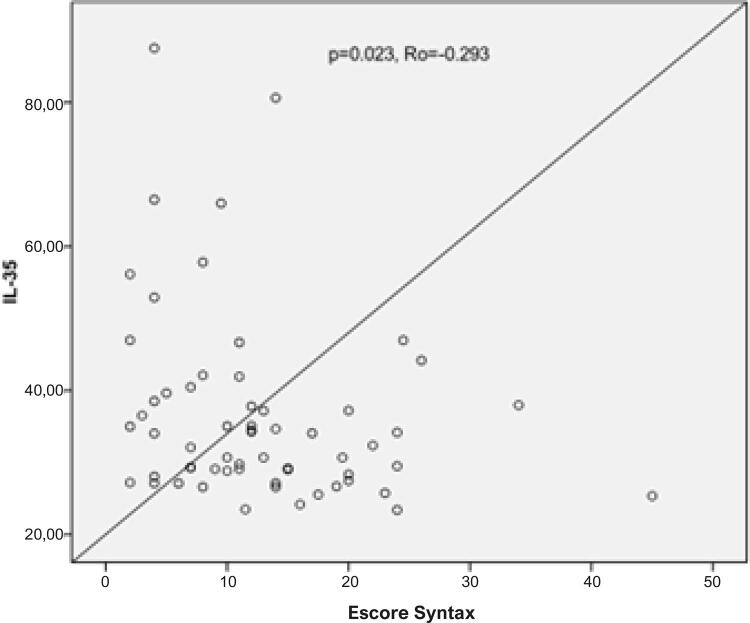
– Associação entre níveis de IL-35 e o escore Syntax.

**Figura 3:**
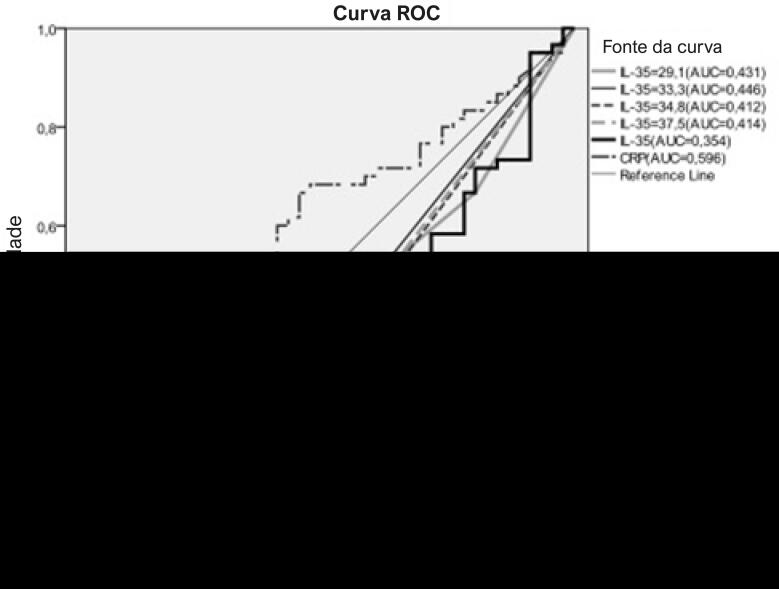
– Poder diagnóstico e pontos de corte de valores de IL-35 e PCR em relação à presença da DAC (análise ROC).

Os aspectos demográficos dos grupos de pacientes e controle estão demonstados na Tabela 1. Da mesma forma, a idade média do grupo de pacientes foi maior do que no grupo controle. Gênero, hábito de fumar, DM, HT, HL e IMC foram semelhantes entre os grupos (Tabela 1). Os achados laboratoriais dos grupos estão demonstrados na Tabela 2. Os níveis de IL-35 foram significativamente menores no grupo de pacientes em relação ao grupo controle (p=0,008) (Figura 1). Além disso, enquanto a contagem de leucócitos, níveis de colesterol total e lipoproteína de baixa densidade (LDL) estavam maiores no grupo de pacientes, a contagem de trombócitos foi mais baixa no grupo de pacientes em comparação ao grupo controle (Tabela 2). A Tabela 3 ilustra os níveis de IL-35 de acordo com os principais aspectos demográficos e laboratoriais no grupo de pacientes. Da mesma forma, os níveis de IL-35 foram significativamente mais baixos em indivíduos com diabetes no grupo de pacientes (p=0,042). Analisamos mais profundamente os níveis de IL-35 em ambos os grupos, em pacientes com e sem diabetes, para demonstrar se os níveis baixos de IL-35 em pacientes com DAC estavam relacionados à diabetes ou não (Tabela 4). Os níveis de IL-35 não estavam associados à presença de diabetes (p=0,18). Embora os níveis de IL-35 tenham sido semelhantes entre os pacientes com DAC com SS baixo (<22) e alto (≥22), foram menores em pacientes com DAC com GS alto (≥20) do que entre aqueles com GS baixo (<20) (p=0,51 e p=0,043, respectivamente) (Tabela 5). A análise de correlação entre IL-35 e outros parâmetros está demonstrada na Tabela 6. Os níveis de IL-35 tinham uma correlação negativa leve com o SS e com os níveis de colesterol total (p=0,036) (Figura 2) (Tabela 6). Por fim, na análise de regressão logística que incluía IL-35, HM, gênero, idade, HT, DM tipo 2 e hábito de fumar, somente a DM tipo 2 (p= 0,049, RR= 3,44, CI: 1,004-11,8), o hábito de fumar (p <0,001, RR= 11,27, IC: 3,45-36,83) e os níveis de IL-35 (p= 0,017, RR= 1,02, GA: 1,005 -1,053) tiveram um efeito independente na presença da DAC (Tabela 7). O ponto de corte para os níveis de IL-35 para detectar a DAC foi a avaliado pela análise ROC (Figura 3) (Tabela 8).

**Tabela 1:** – Aspectos demográficos dos grupos de pacientes e controles

	Paciente, n(%)	Controle, n(%)	Valor de p
Total	60(100)	46(100)	
Idade (média±DP)	59±9,1	54,5(8,9)	0,013*
**Sexo**			
Masculino	42(70)	28(60,9)	0,32
Feminino	18(30)	18(39,1)	
**Fumar**	38(63,3)	12(26,1)	<0,001
**Diabetes**	22(36,7)	10(21,7)	0,09
**Hipertensão**	38(63,3)	21(45,7)	0,07
**Hiperlipidemia**	16(26,7)	9(19,6)	0,008
**IMC (média±DP)**	26,3±4,4	28.2±3,8	0,07*

Teste de qui-quadrado, *teste t de Student; IMC: índice de massa corporal.

**Tabela 2:** – Achados laboratoriais nos grupos de pacientes e controles

	Grupo de pacientes	Grupo controle	Valor de p
IL-35 (pg/ml)	Mediana: 33,2 Intervalo: 23,39-172,6	Mediana: 36,9 Intervalo: 23,39-238,1	**0,008**
Creatinina (mg/dl)	Mediana: 0,8 Intervalo: 0,35-1,1	Mediana: 0,8 Intervalo: 0,42-1,2	0,43
Colesterol LDL (mg/dl)(média+DP)	149±39,3	116,5±35,9	<0,001*
Colesterol total (mg/dl) (média+DP)	229,5±47,1	190,5±46,3	<0,001*
Colesterol HDL (mg/dl)	Mediana: 44 Intervalo: 25-72	Mediana: 45 Intervalo: 27-72	0,98
Triglicérides (mg/dl) (média+DP)	150,5±81	115,5±83,2	0,19*
Contagem de leucócitos (10^3^/mm^3^) (média+DP)	8250±2670	6550±2030	0,006*
Hematócrito (%)	Mediana: 40,8 Intervalo: 29,7-52,1	Mediana: 40,9 Intervalo: 28,6-50,2	0,93
Hemoglobina (g/dl) (média+DP)	13,9±1,7	13,4±1,8	0,37*
Contagem de trombócitos (10^3^/mm^3^) (média+DP)	248±104	270±64,7	0,015*
		Mediana: 2,6	0,09
PCR-as (mg/L)	Mediana: 4,1 Intervalo: 0,32-18,2	Intervalo: 0,2-17,8	
HbA1C(%)	Mediana: 5,9 Intervalo: 4,0-13,2	Mediana: 5,8 Intervalo: 4,5-7,6	0,45

IL-35: Interleucina-35; LDL: lipoproteína de baixa densidade; HDL: lipoproteína de alta densidade; PCR-as: proteína C reativa de alta sensibilidade; HbA1C: Hemoglobina A1C. Teste U de Mann-Whitney, *teste t de Student

**Tabela 3:** – Níveis de IL-35 de acordo com os principais aspectos demográficos e laboratoriais no grupo de pacientes

	Mediana/Intervalo Interquartil	Valor de p
Sexo		0,55
Masculino	Mediana: 34,7 Intervalo: 23,39-103,9	
Feminino	Mediana: 34,3 Intervalo: 26,5-172,6	
Idade		0,12
<50	Mediana: 38,1 Intervalo: 27,2-172,6	
≥50	Mediana: 34,7 Intervalo: 23,9-133,3	
Hipertensão (+)	Mediana: 34 Intervalo: 23,39-172,6	0,11
(-)	Mediana: 35,8 Intervalo: 25,4-80,61	
Diabetes (+)	Mediana: 30,4 Intervalo: 23,9-172,6	0,042
(-)	Mediana: 35,0 Intervalo: 24,16-133,3	
Fumar (+)	Mediana: 35,0 Intervalo: 27,11-133,3	0,5
(-)	Mediana: 34,1 Intervalo: 23,39-172,6	
Hiperlipidemia (+)	Mediana: 34,6 Intervalo: 25,4-133,3	0,56
(-)	Mediana: 34,1 Intervalo: 23,39-172,6	
IMC		0,71
IMC<25	Mediana: 34,8 Intervalo: 24,16-56,12	
IMC≥25	Mediana: 34,2 Intervalo: 23,9-172,6	
IMC		0,13
IMC<30	Mediana: 35 Intervalo: 25,3-172,6	
IMC≥30	Mediana: 30,7 Intervalo: 23,9-87,6	
PCR-as		0,61
<5mg/L	Mediana: 34,6 Intervalo: 24,16-87,51	
≥5mg/L	Mediana: 33,4 Intervalo: 23,9-172,6	

*IMC: índice de massa corporal; PCR-as: proteína C reativa de alta sensibilidade.*

**Tabela 4:** – Níveis de IL-35 de acordo com o histórico de diabetes em ambos os grupos

	Variáveis	DAC(+)	DAC(-)	p
DM(-)	IL-35	Mediana: 34,4 Intervalo: 24,16-133,3	Mediana: 40,6 Intervalo: 24,59-238,1	0,021
DM(+)	IL-35	Mediana: 29,6 Intervalo: 23,39-172,6	Mediana: 33,4 Intervalo: 25,09-199,1	0,18

*DAC: doença arterial coronariana*

**Tabela 5:** – Níveis de IL-35 no grupo de pacientes de acordo com os escores Gensini e Syntax

		Número de pacientes (%)	IL-35(média±DP)	Valor de p
Escore Gensin	<20	23(38,3)	35±17,4	0,043*
	≥20	37(61,7)	30,7±8,6	
Escore Syntax	<22	52(86,7)	33,2±13,7	0,51^¥^
	≥22	8(13,3)	31,8±8,9	

* Teste t de Student, ¥ Teste U de Mann-Whitney.

**Tabela 6:** – Análise de correlação entre a IL-35 e outros parâmetros

Variáveis		Rho	Valor de p	Variáveis		Rho	Valor de p
IL-35	Colesterol total	-0,204	0,036	IL-35	Escore Gensini	-0,208	0,11
IL-35	Leucócitos	0,12	0,2	IL-35	Escore Syntax	-0,293	0,023
IL-35	PCR-as	-0,03	0,75				

*PCR-as: proteína C reativa de alta sensibilidade; Rho: coeficiente de correlação de teste de Spearman ou ρ de Spearman.*

**Tabela 7:** – Análise de regressão logística dos principais parâmetros para previsão da DAC

Parâmetros	RR	IC95%	Valor de p
Hiperlipidemia	1,22	0,34-4,34	0,75
Sexo	0,9	0,27-2,98	0,87
Idade	0,96	0,91-1,02	0,23
Hipertension	17	0,56-5,15	0,34
Diabetes	3,44	1,004-11,8	0,049
Fumar	11,27	3,45-36,83	<0,001
IL-35	1,02	1,005-1,053	0,017

*RR: razão de risco; IC: intervalo de confiança; DAC: doença arterial coronariana.*

**Tabela 8:** – Valores da Área sob a Curva (AUC) para IL-35 e pontos de corte substitutos

	AUC	SE	IC95%	p
IL-35	0,354	0,056	0,24-0,46	**0,011**
Ponto de corte=29,1	0,431	0,056	0,32-0,54	0,22
Ponto de corte=33,3	0,446	0,056	0,33-0,55	0,33
Ponto de corte=34,8	0,412	0,056	0,3-0,52	0,14
Ponto de corte=37,5	0,414	0,056	0,3-0,52	0,11

## Discussão

Sabe-se que a aterosclerose é multifatorial e está muito relacionada à inflamação.^4^ O equilíbrio entre as citocinas proinflamatórias e anti-inflamatórias está associado à estabilidade das placas e à progressão da aterosclerose.^4,5^ Baixos níveis de IL-35 são indicativos tanto da resposta anti-inflamatória insuficiente quanto do nível da inflamação na DAC.^18,19^ Normalmente, a IL-35 só é induzida com inflamação, e depois detectada no sangue periférico.^19^ Em um estudo conduzido com ratos, a IL-35 não foi detectada no perfil tecidual de sujeitos saudáveis; porém, estava aumentada em amostras teciduais de ratos com resposta inflamatória formada externamente.^20^

A expressão de IL-35 e os níveis sanguíneos foram avaliados por muitos pesquisadores em cenários clínicos diferentes.^20-23^ Kempe et al.,^21^ demonstraram forte expressão de IL-35 nas células endoteliais, células do músculo liso vascular e macrófagos de pacientes sintomáticos com placas nas carótidas, enquanto não poderiam detectar a expressão de IL-35 na camada íntima da carótida saudável.^21^

Por outro lado, há uma relação linear entre a gravidade da inflamação e a baixa IL-35 na DAC.^21^ A resposta anti-inflamatória inadequada na aterosclerose leva à resposta inflamatória excessiva na placa.^21^ Em outras palavras, quanto mais baixos os níveis de IL-35, mais alta a carga aterosclerótica nas artérias coronárias.^22^ Da mesma forma, Yanmei et al. demonstraram uma correlação entre níveis decrescentes de IL-35 e a gravidade da doença inflamatória intestinal.^22^ Esses dados sugerem que, primeiro, a IL-35 aumenta de forma secundária à inflamação, e depois diminui conforme a inflamação se torna mais grave.^22^

No grupo de pacientes, os níveis de IL-35 foram significativamente mais baixos em pacientes com GS alto em comparação àqueles com GS baixo (35±17,4 vs. 30,7±8,6, p=0,043). Por outro lado, os níveis de IL-35 foram semelhantes entre pacientes com CAD com SS baixo e alto (31,8±8,9 vs. 33,2±13,7, p=0,51); porém, em nossa opinião, o número de pacientes com SS alto foi muito pequeno para uma análise significativa. Um motivo para incluirmos poucos pacientes com SS alto pode ser o fato de termos consideraedo somente pacientes com DAC estável, e não indivíduos com síndrome coronária aguda nem aqueles com histórico de DAC. Nossos achados são únicos porque, até onde sabemos, este estudo é o primeiro que revelou uma correlação entre a gravidade da DAC e os níveis de IL-35. Recentemente, Lin et al.,^18^ demonstraram níveis mais baixos de IL-35 em pacientes com DAC estável e infarto agudo do miocárdio (IAM) em comparação ao grupo controle; porém, não avaliaram o efeito da extensão e gravidade da DAC.^18^ Os níveis mais baixos de IL-35 estão no grupo IAM do estudo de Lin.^18^ Assim, especulou-se que a IL-35 pode ser usada como preditor do desfecho clínico para aterosclerose.^19^

Como o teste PCR-as é um marcador inflamatório conhecido para aterosclerose, é considerado como preditor independente da DAC.^23^ Embora os níveis de PCR-as sejam mais altos no grupo de pacientes do que no controle, não foram estatisticamente significativos em nosso estudo. Além disso, não houve correlação entre a PCR-as e a IL-35. Este achado pode parecer incompatível com a literatura, porém, em nossa opinião, isso pode se dever ao fato de que a PCR-as está mais relacionada à instabilidade da placa e associada a eventos cardiovasculares, e não à extensão da aterosclerose. Assim, a IL-35 pode pode ser um marcador melhor do que a PCR-as para DAC estável.

Além disso, Wang et al.,^19^ conduziram um estudo *in vitro* com animais e fez com que as células T regulatórias de ratos secretassem IL-35 para tratar a doença inflamatória intestinal e artrite induzida pelo colágeno, conhecidas por estarem relacionadas à inflamação crônica.^19^ Assim, a regulação celular da expressão de IL-35 pode ser um novo tratamento para DAC.

A principal limitação do nosso estudo foi seu desenho em centro único e o número relativamente baixo de participantes. Em segundo lugar, só avaliamos a DAC por meio da AGC; porém, outros métodos, como o ultrassom intravascular e a tomografia de coerência óptica, que são capazes de determinar a morfologia da placa, aumentariam o poder deste estudo. Por fim, o estudo seria mais completo se pudéssemos medir outras citocinas inflamatórias, como fator de crescimento tumoral beta e interleucina-10; porém, tínhamos um orçamento limitado.

## Conclusão

A IL-35 é uma nova citocina que tem efeitos imunossupressores e anti-inflamatórios.^24^ Os principais achados deste estudo são os níveis baixos de IL-35 em pacientes com DAC estável, principalmente com GS alto, e a correlação negativa entre IL-35 e SS. Esses achados sugerem que os níveis baixos de IL-35 estão associados à extensão e à gravidade de DAC. Além disso, a secreção de IL-35 externamente induzida pode ser usada para o tratamento de aterosclerose. Mais estudos devem ser realizados em populações maiores.
