# Supplementary material for: Níveis de Interleucina-35 em Pacientes com Doença Arterial Coronariana Estável
Source: Arq Bras Cardiol. 2021 Dec 8;118(2):400–8. [Article in Portuguese] doi: 10.36660/abc.20200945 (PMC8856683; doi:10.36660/abc.20200945)
Supplement: Translation

# Translation: Interleukin-35 Levels in Patients with Stable Coronary Artery Disease

**Authors:** Ersan Oflar, Mustafa Hakan Sahin, Bulent Demir, Abdulcelil Sait Ertugrul, Didem Melis Oztas, Metin Onur Beyaz, Murat Ugurlucan, Fatma Nihan Turhan Caglar

**Affiliations:** 1 Turkey Bakirkoy Dr Sadi Konuk Training and Research Hospital, Department of Cardiology, Istambul - Turkey; 2 Turkey Akdeniz Saglik Yasam Vakfi, Department of Cardiology, Antalya - Turkey; 3 Qatar Turkish Hospital, Department of Cardiology, Doha - Qatar; 4 Turkey Bagcilar Training and Research Hospital, Department of Cardiovascular Surgery, Istambul - Turkey; 5 Turkey Istanbul Medipol University Faculty of Medicine, Department of Cardiovascular Surgery, Istambul - Turkey

**Keywords:** Coronary artery disease, Atherosclerosis, Interleukin-35, Gensini score, Syntax score

## Abstract

**Background:**

It has been shown that interleukin-35 (IL-35) subunits are strongly expressed in atherosclerotic plaques in humans. Therefore, it is considered to play a role in atherosclerosis.

**Objectives:**

In this study, IL-35 levels were compared with the control group in patients with stable coronary artery disease (CAD), and the association between IL-35 levels and the lesion type, lesion severity and extension was investigated with the Gensini score (GS) and the Syntax score (SS) in the patient group.

**Methods:**

Sixty patients (18 female and 42 male) with CAD diagnosed by coronary angiography, who presented with typical chest pain and positive noninvasive cardiac stress test, and 46 patients (18 female and 28 male) with normal coronary lumenogram, were included in this study. Gensini and Syntax scores were calculated in the patient group, and these values were compared with IL-35 levels. Non-normally distributed variables were analyzed by the Mann-Whitney U test, whereas normally distributed parameters were assessed by Student’s t-test. The difference between categorical variables were evaluated by the Chi-square or Fisher test. P-values<0.05 were considered as statistically significant.

**Results:**

No significant differences were observed between patients and the control group in terms of demographic characteristics and laboratory findings. Compared to the control group, IL-35 levels of the CAD group were considerably lower (36.9±63.9 ng/ml vs. 33.2±13.2 ng/ml, p<0.008). Although not statistically significant, IL-35 levels were higher in patients with low SS than among those with high SS (33.2±13.7 vs. 31.8±8.9, p=0.51). The IL-35 values of the patients with high GS were significantly lower than in patients with low GS (35±17.4 vs. 30.7±8.6, p=0.043).

**Conclusion:**

It has been shown that IL-35 levels can be a new biomarker for stable CAD, and IL-35 is associated with the extension of CAD.

## Introduction

Coronary artery disease (CAD) is a leading cause of death worldwide, although the prevalence of CAD mortality has recently decreased in Europe and in the United States.^1,2^ CAD is a progressive and systemic disorder mainly caused by atherosclerosis.^3^

Although inflammation plays a major role in the development of atherosclerosis, the precise underlying mechanism is still not clear.^4^ Anti-inflammatory cytokines such as interleukin-10 (IL-10) and transforming growth factor-1 (TGF-1) are widely evaluated in atherosclerosis trials.^5-11^ Recently published studies have demonstrated that while low IL-10 and TGF-1 levels are associated with the progression of atherosclerosis and the development of acute coronary syndrome, high IL-10 and TGF-1 levels are correlated with good prognosis in CAD.^5-11^

Interleukin-35 (IL-35), a recently defined anti-inflammatory cytokine, supresses the CD4+T cell activity, induces the production of regulatory T cells, and reduces the progression of inflammatory and autoimmune diseases; hence, it also plays a role in atherosclerosis.^12-15^

The aim of this study was to evaluate IL-35 plasma levels in patients with stable CAD, and the association between IL-35 and CAD severity and extension using the Syntax (SS) and Gensini (GS).

## Methods

This is a cross-sectional, observational study carried out in a tertiary referral center. One hundred and six consecutive patients who underwent diagnostic coronary angiography in Bakırköy Dr. Sadi Konuk Research and Training Hospital Cardiology Clinic between July 2018 and May 2019 were included in the study. The patient group consisted of 60 patients (42 male, 18 female) with stable CAD who presented with epicardial coronary artery stenosis of more than 50% in the coronary angiogram (CAG). The control group consisted of 46 patients (28 male, 18 female) who had chest pain, but normal CAG. All of the patients had undergone modified objective ischemia assessment, and they all had positive test results in terms of ischemia. The required number of patients was decided based on previous studies.^7^ The patients who were appropriate for the study according to inclusion criteria were included until we reached the necessary number of patients between July 2018 and May 2019.

A previous diagnosis of diabetes mellitus (DM), use of anti-diabetic medicines, or a fasting glucose level of 126 mg/dl on two occasions in previously untreated patients were required to diagnose DM. Hypertension (HT) was defined based on the previous use of antihypertensive medications, systolic pressure higher than 140 mmHg, or diastolic pressure higher than 90mm Hg in at least two separate measurements. The glomerular filtration rate (GFR) was estimated by using the Modification of Diet in Renal Disease (MDRD) equation at admission. Body mass index (BMI) was calculated according to the World Health Organization (WHO) criteria. Hyperlipidemia (HL) was defined based on the previous use of antilipidemics in the 6 previous months, or high serum levels of lipids measured after 8 hours of fasting [low-density lipoprotein (LDL) >160 mg/dl, total cholesterol (TC) >240 mg/dl, or triglycerides (TG) >160 mg/dl)].

All patients signed an informed consent and this study is in accordance with the Declaration of Helsinki. Our study was approved by the local Institutional Review Board and Ethics Commitee.

Patients with known CAD, estimated glomerular filtration rate (eGFR) <60ml/min, those with valvular disease, individuals with systolic blood pressure higher than 140 mmHg and diastolic blood pressure higher than 90 mmHg despite the treatment, heart failure, liver failure, acute/chronic infection, fever, muscle aches, headache, the ones receiving antibiotic therapy, individuals with immunoproliferative disease, rheumatic disease, cancer, osteoporosis and the ones who were older than 75 years were excluded.

### Objective ischemia assessment

All of the patients had undergone a non-invasive stress test for ischemia assessment. Mostly, the modified Bruce exercise test was performed. At least 1 mm horizontal or downsloping ST segment depression in ≥2 derivations after 60*80 msec from the J point during exercise was considered as an abnormal test. The Duke treadmill score was used for risk stratification.^11^ CAG was performed in patients who had medium or high Duke treadmill score. Myocardial perfussion scintigraphy (MPS) was used to evaluate ischemia in inpatients who had left the bundle branch block or ≥1mm ST depression in the at rest electrocardiogram (ECG), nondiagnostic exercise test, or wth poor exercise capacity. CAG was performed in patients who had medium or high ischemia level on MPS.

### Biomarker measurements

All of the patients’ laboratory data, such as cardiac troponin-T (cTr-T), creatinine, white blood cell count (WBC), high sensitive C-reactive protein (hsCRP) etc, were documented.

Blood samples for IL-35 were drawn in the catheterization laboratory before coronary angiography in all of the participants. Blood samples were obtained by venipuncture in ethylenediaminetetraacetic acid (EDTA) blood collection tubes without additives, and immediately centrifuged at 4000 rpm for 10 minutes. The sample was collected aftercentrifugation and stored at -80 ºC until analysis (no longer than 6months). The samples were thawed only once. The biotin-based double antibody sandwich technique using the enzyme-linked immune sorbent assay (ELISA) kit was used for the analysis of IL-35 (Human Interleukin 35: Yehua Biological Technology; Cat No:YHB1739Hu). IL-35 was added to wells that were precoated with IL-35 monoclonal antibody and, then, incubated. After incubation, anti-IL-35 antibodies labeled with biotin were added to the unit with streptavidin-HRP to form the immune complex. After washing, unbound enzymes were removed, then added to substrates A and B. The solution turned blue and changed to yellow with the effect of acid. The shades of the solution and the concentration of IL-35 were positively correlated. Results were expressed as ng/ml. Intra-assay and inter-assay coefficients of variation (CV) of the analysis were <10% and <12%, respectively.

### Coronary angiogram

All CAG procedures were performed using the standard Judkins method with a cineangiography device (Axiom Artis, Siemens, Germany). All of the angiograms were recorded in compact discs, in DICOM format, and visually examined by two experienced interventional cardiologists blinded to the study. The severity and extent of CAD were evaluated according to the SS and GS. The degree of lumen narrowing, concentricity and eccentricity of the plaques were evaluated. According to the Gensini Score system, 1 point is given for 1–25% stenosis; 2 points for 26–50%; 4 points for 51–75%; 8 points for 76–90%; 16 points for 91–99%; and 32 points for 100% stenosis. Then, the number of points for each lesion is multiplied by the coefficient which is given for each main vascular segment according to the functional significance of the vessel (left main coronary artery × 5; proximal segment of the left anterior descending coronary artery (LAD) × 2.5; proximal segment of the circumflex artery × 2.5; mid-segment of LAD × 1.5; right coronary artery, distal segment of LAD, posterolateral artery and the obtuse marginal artery × 1; and others × 0.5), and the sum of all points constitutes the total score.^16^ Scoring was performed together with two observers and the average value. GS < 20 was considered as mild CAD (group 1), and GS ≥ 20 was accepted as severe CAD (group 2). The SS corresponding to the lesion complexity was measured by the coronary tree characteristics, as well as the lesion locations and specifics.^17^ The score is measured using the openly accessible web based score calculator (www.syntaxscore.com). Scorings were performed and averaged by two observers who were blinded to the study groups.

### Statistical Analysis

Statistical analysis was performed using the software SPSS, version 16. The normal distribution of continuous variables was analyzed by visual (histogram) and analitic methods (Kolmogorov-Smirnov). Continuous variables with normal distribution were shown as mean±standard deviation (SD). Continuous variables with non-normal distribution were demonstrated as median and interquartile range. Non-normally distributed variables were analyzed by the Mann-Whitney U test, whereas normally distributed parameters were assessed by the unpaired Student’s t-test. The differences between categorical variables were evaluated by Chi-square or Fisher test. The association between non-normally distributed variables were analyzed by the Spearman test; on the other hand, the Pearson test was used for the correlation between normally distributed variables. The chi-square test was used for sensitivity, specificity, negative, and positive predictive values. The logistic regression analysis was performed to demonstrate IL-35 as an independent CAD risk factor among traditional CAD risk factors. The effectiveness and compatibility of the created model was tested by the Hosmer-Lemeshow test. P-values <0.05 were considered as statistically significant.

## Results

The demographic features of the patient and control groups are shown in Table 1. Accordingly, the mean age of the patient group was higher than that in the control group. Gender, smoking, DM, HT, HL, and BMI were similar between groups (Table 1). Laboratory findings of the groups are demonstrated in Table 2. IL-35 levels were significantly lower in the patient group than in the control group (p=0.008) (Figure 1). Besides, while leucocyte count, total cholesterol, and low density lipoprotein (LDL) levels were higher in the patient group, thrombocyte count was lower in the patient group compared to the control group (Table 2). Table 3 illustrates IL-35 levels according to main demographic and laboratory features in the patient group. Likewise, IL-35 levels were significantly lower in individuals with diabetes in the patient group (p=0.042). We further analyzed IL-35 levels in both groups, in patients with and without diabetes, in order to demonstrate if the low IL-35 levels in patients with CAD were owed to diabetes or not (Table 4). IL-35 levels were not associated with the presence of diabetes (p=0.18). Although IL-35 levels were similar among CAD patients with low (<22) and high SS (≥22), it was lower in CAD patients with high (≥20) than among those with low (<20) GS (p=0.51 and p=0.043, respectively) (Table 5). The correlation analysis between IL-35 and other parameters is shown in Table 6. IL-35 levels had a mild negative correlation with the SS and total cholesterol levels (p= 0.036) (Figure 2) (Table 6). Finally, in the logistic regression analysis that included IL-35, HM, gender, age, HT, type 2 DM, and smoking, only type 2 DM (p= 0.049, RR= 3.44, CI: 1.004-11.8), smoking (p <0.001, RR= 11.27, CI: 3.45-36.83) and IL-35 levels (p= 0.017, RR= 1.02, GA: 1.005 -1.053) were found to have an independent effect on the presence of CAD (Table 7). The cut-off point for IL-35 levels for detecting CAD was evaluated by the ROC analysis (Figure 3) (Table 8).

**Table 1:** – Demographic features of the patient and control groups

	Patient, n(%)	Control, n(%)	p-value
Total	60(100)	46(100)	
Age (mean±SD)	59±9.1	54.5(8.9)	0.013*
**Sex**			
Male	42(70)	28(60.9)	0.32
Female	18(30)	18(39.1)	
**Smoking**	38(63.3)	12(26.1)	<0.001
**Diabetes**	22(36.7)	10(21.7)	0.09
**Hypertension**	38(63.3)	21(45.7)	0.07
**Hyperlipidemia**	16(26.7)	9(19.6)	0.008
**BMI (mean±SD)**	26.3±4.4	28.2±3.8	0.07*

Chi-square test, *Student’s t- test; BMI:body mass index.

**Table 2:** – Laboratory findings of the patient and control groups

	Patient group	Control group	p-value
IL-35 (pg/ml)	Median: 33.2 Range: 23.39-172.6	Median: 36.9 Range: 23.39-238.1	**0.008**
Creatinine (mg/dl)	Median: 0.8 Range: 0.35-1.1	Median: 0.8 Range: 0.42-1.2	0.43
LDL cholesterol (mg/dl)(mean+SD)	149±39.3	116.5±35.9	<0.001*
Total cholesterol (mg/dl) (mean+SD)	229.5±47.1	190.5±46.3	<0.001*
HDL cholesterol (mg/dl)	Median: 44 Range: 25-72	Median: 45 Range: 27-72	0.98
Triglyceride (mg/dl) (mean+SD)	150.5±81	115.5±83.2	0.19*
Leucocyte count (103/mm3) (mean+SD)	8250±2670	6550±2030	0.006*
Hematocrit (%)	Median: 40.8 Range: 29.7-52.1	Median: 40.9 Range: 28.6-50.2	0.93
Hemoglobin (g/dl) (mean+SD)	13.9±1.7	13.4±1.8	0.37*
Thrombocyte count(103/mm3) (mean+SD)	248±104	270±64.7	0.015*
hs-CRP (mg/L)	Median: 4.1 Range: 0.32-18.2	Median: 2.6 Range: 0.2-17.8	0.09
HbA1C(%)	Median: 5.9 Range: 4.0-13.2	Median: 5.8 Range: 4.5-7.6	0.45

IL-35: Interleukin 35; LDL:low density lipoprotein; HDL:high density lipoprotein; hs-CRP: high sensitive C-reactive protein; HbA1C:Hemoglobin A1C. Mann-Whitney U-test, *Student’s t-test.

**Figure 1:** – IL-35 levels in the patient and control groups (Box-plot).

**Table 3:** – IL-35 levels according to the main demographic and laboratory features in the patient group

	Median/Interquartile Range	p-value
Sex		0.55
Male	Median: 34.7 Range: 23.39-103.9	
Female	Median: 34.3 Range: 26.5-172.6	
Age		0.12
<50	Median: 38.1 Range: 27.2-172.6	
≥50	Median: 34.7 Range: 23.9-133.3	
Hypertension (+)	Median: 34 Range: 23.39-172.6	0.11
(-)	Median: 35.8 Range: 25.4-80.61	
Diabetes (+)	Median: 30.4 Range: 23.9-172.6	0.042
(-)	Median: 35.0 Range: 24.16-133.3	
Smoking (+)	Median: 35.0 Range: 27.11-133.3	0.5
(-)	Median: 34.1 Range: 23.39-172.6	
Hyperlipidemia (+)	Median: 34.6 Range: 25.4-133.3	0.56
(-)	Median: 34.1 Range: 23.39-172.6	
BMI		0.71
BMI<25	Median: 34.8 Range: 24.16-56.12	
BMI≥25	Median: 34.2 Range: 23.9-172.6	
BMI		0.13
BMI<30	Median: 35 Range: 25.3-172.6	
BMI≥30	Median: 30.7 Range: 23.9-87.6	
hs-CRP		0.61
<5mg/L	Median: 34.6 Range: 24.16-87.51	
≥5mg/L	Median: 33.4 Range: 23.9-172.6	

*BMI: body mass index; hs-CRP: high sensitive C-reactive protein.*

**Table 4:** – IL-35 levels according to history of diabetes in both groups

	Variables	CAD(+)	CAD(-)	p
DM(-)	IL35	Median: 34.4 Range: 24.16-133.3	Median: 40.6 Range: 24.59-238.1	0.021
DM(+)	IL35	Median: 29.6 Range: 23.39-172.6	Median: 33.4 Range: 25.09-199.1	0.18

*CAD: Coronary Artery Disease*

**Table 5:** – IL-35 levels in the patient group according to the Gensini and Syntax scores

		Patient number (%)	IL-35(mean±SD)	p-value
Gensini score	<20	23(38.3)	35±17.4	0.043*
	≥20	37(61.7)	30.7±8.6	
Syntax score	<22	52(86.7)	33.2±13.7	0.51¥
	≥22	8(13.3)	31.8±8.9	

* Student’s t-test, ¥ Mann Whitney U-test.

**Table 6:** – Correlation analysis between IL-35 and other parameters

Variables		Rho	p- value	Variables		Rho	p- value
IL35	Total cholesterol	-0.204	0.036	IL35	Gensini score	-0.208	0.11
IL35	Leucocyte	0.12	0.2	IL35	Syntax score	-0.293	0.023
IL35	hs-CRP	-0.03	0.75				

*hs-CRP: high sensitive C-reactive protein; Rho: Spearman’s rank correlation coefficient or Spearman’s ρ.*

**Figure 2:** – The association between IL-35 levels and Syntax score.

**Table 7:** – Logistic regression analysis of main parameters in CAD prediction

Parameters	RR	%95CI	p-value
Hyperlipidemia	1.22	0.34-4.34	0.75
Sex	0.9	0.27-2.98	0.87
Age	0.96	0.91-1.02	0.23
Hypertension	17	0.56-5.15	0.34
Diabetes	3.44	1.004-11.8	0.049
Smoking	11.27	3.45-36.83	<0.001
IL-35	1.02	1.005-1.053	0.017

*RR: risk ratio; CI: confidence interval; CAD: Coronary Artery Disease.*

**Figure 3:** – Diagnostic power and potential cut-off values of IL-35 and CRP values in terms of the presence of CAD (ROC analysis).

**Table 8:** – Area Under Curve (AUC) values for IL-35 and surrogate cut-off values

	AUC	SE	95%CI	p
IL35	0.354	0.056	0.24-0.46	**0.011**
Cut-off value=29.1	0.431	0.056	0.32-0.54	0.22
Cut-off value=33.3	0.446	0.056	0.33-0.55	0.33
Cut-off value=34.8	0.412	0.056	0.3-0.52	0.14
Cut-off value=37.5	0.414	0.056	0.3-0.52	0.11

## Discussion

It is well-known that atherosclerosis is multifactorial and closely related with inflammation.^4^ The balance between proinflammatory and antiinflammatory cytokines is associated with plaque stabilisation and atherosclerosis progression.^4,5^ Low IL-35 levels are indicators of both insufficient antiinflammatory response and the amount of inflammation in CAD.^18,19^ Normally, IL-35 is only induced with inflammation, and then detected in peripheral blood.^19^ In a study conducted with mice, IL-35 was not detected in the tissue profile of healthy subjects; however, it was increased in tissue samples of mice with externally formed inflammatory response.^20^

IL-35 expression and blood levels have been evaluated by many researchers in different clinical scenarios.^20-23^ Kempe et al.^21^ demonstrated strong expression of IL-35 in endothelial cells, vascular smooth muscle cells, and macrophages of symptomatic patients with carotid plaques, whereas they could not detect IL-35 expression in healthy carotid intima.^21^

On the other hand, there is a linear relationship between the severity of inflammation and low IL-35 in CAD.^21^ Inadequate antiinflammatory response in atherosclerosis leads to excessive inflammatory response in the plaque.^21^ In other words, the lower the IL-35 levels, the higher the atherosclerotic burden in the coronary arteries.^22^ Similarly, Yanmei et al. demonstrated a correlation between decreasing IL-35 levels and the severity of inflammatory bowel disease.^22^ These data suggest that, first, IL-35 rises secondary to the inflammation, and then declines as the inflammation gets severe.^22^

In the patient group, IL-35 levels were significantly lower in patients with high GS in comparison with those with low GS (35±17.4 vs . 30.7±8.6 , p= 0.043). Conversely, IL-35 levels were similar among CAD patients with low and high SS (31.8±8.9 vs. 33.2±13.7, p=0.51); however, in our opinion, the number of patients with high SS was too low for a powerful analysis. A reason for having few patients with high SS might be because we only enrolled patients with stable CAD, and not individuals with acute coronary syndrome nor those with a history of known CAD. Our findings are unique because, to our knowledge, this study is the first that revealed a correlation between CAD severity and IL-35 levels. Recently, Lin et al.^18^ showed lower IL-35 levels in patients with stable CAD and acute myocardial infarction (AMI) in comparison to the control group; however, they did not evaluate the effect of CAD extent and severity.^18^ The lowest IL-35 levels were in the AMI group in Li’s study.^18^ Hence, it it has been speculated that IL-35 may be used as a clinical outcome predictor in atherosclerosis.^19^

An hs-CRP test is a well-known inflammatory marker in atherosclerosis, and is considered as an independent predictor of CAD.^23^ Although hs-CRP levels were higher in the patient group than among controls, it was not statistically significant in our study. Furthermore, there was no correlation between hs-CRP and IL-35. This finding may seem incompatible with the literature, however, in our opinion, it may be because hs-CRP is more related with plaque instability and its associated cardiovascular events, rather than the extent of atherosclerosis. Therefore, IL-35 might be a better marker than hs-CRP in stable CAD.

Moreover, Wang et al.^19^ conducted an *in vitro* animal study, and made regulatory T cells’ of mice secrete IL-35 to treat inflammatory bowel disease and collagen-induced arthritis, which are known to be related with chronic inflammation.^19^ Thus, the cellular regulation of IL-35 expression may be a new treatment target in CAD.

The main limitation of our study was its single-center design and relatively low number of participants. Secondly, we only evaluated CAD by CAG; however, other methods, such as intravascular ultrasound and optical coherence tomography, which are capable of determining plaque morphology, would increase the power of this study. Lastly, the study would be more thorough if we could measure other inflammatory cytokines, like tumor growth factor beta and interleukin-10; yet, we had a limited budget.

## Conclusion

IL-35 is a new cytokine which has immunosuppressive and strong antiinflammatory effects.^24^ The main findings of this study are low IL-35 levels in patients with stable CAD, especially with high GS, and the negative correlation between IL-35 and SS. These findings suggest that low IL-35 levels are associated with the extent and severity of CAD. Additionally, externally induced IL-35 secretion might be used in atherosclerosis treatment. Further studies conducted in larger populations are required.
